# A Rare Case Presentation: Diagnosing Primary Biliary Cholangitis in a Male Patient With Concomitant Type 1 Diabetes

**DOI:** 10.7759/cureus.16109

**Published:** 2021-07-02

**Authors:** Adejoke M Johnson, Ezekiel J Akpan, Supriya Kale, Anna Patel

**Affiliations:** 1 Medicine, All Saints University School of Medicine, Roseau, DMA; 2 Medicine, All Saints University College of Medicine, Kingstown, VCT; 3 School of Medicine, Windsor University, Canyon, KNA; 4 Gastroenterology and Hepatology, Community First Medical Center, Chicago, USA

**Keywords:** primary biliary cholangitis, autoimmune disease, type 1 diabetes, antimitochondrial antibody, liver cirrhosis, male primary biliary cholangitis, ursodiol, cholestyramine, pruritis

## Abstract

Primary biliary cholangitis (PBC) is characterized as an autoimmune disease that involves the destruction of intrahepatic bile ducts, characteristically leading to a cholestatic liver. The presence of disease-specific antimitochondrial antibodies (AMA) is the gold standard to diagnose PBC. Typically, PBC is known to affect female populations exceedingly over their male counterparts. Associated autoimmune diseases include Sjogren’s and Raynaud’s syndrome, which are also more prevalent in women. The low incidence rates of men affected with PBC, especially with a concomitant type 1 diabetes diagnosis, have resulted in little being known about the clinical course of the disease in this particular population group. Current research suggests no significant histological, serological, or biochemical differences between PBC in males and females. However, some symptoms and clinical associations may be different. This case report presents the rare case of a male patient with type 1 diabetes recently diagnosed with PBC.

## Introduction

Primary biliary cirrhosis was first reported in 1851 by Addison and Gull but later named primary biliary cholangitis (PBC) in 2014 [1,2]. PBC is a chronic autoimmune disorder mediated by T-cell that causes inflammation, scarring, and destruction of the Intrahepatic bile ducts prompting the onset of cholestasis and eventually liver cirrhosis. A vast number of patients diagnosed with PBC do not develop cirrhosis which led to the change in its nomenclature from primary biliary cirrhosis to primary biliary cholangitis in 2014 to better represent the disorder [2]. The precise epidemiology of primary biliary cholangitis is vague as studies in different countries have revealed a vast difference in incidence and prevalence. However, despite the variability in incidence and prevalence in PBC, the prevalence of PBC continues to rise globally at a significant rate. According to a Published study carried out in the USA, from 2006 to 2014, prevalence increased from 21.7 to 39.2 per 100,000 persons. Prevalence increased from 33.5 to 57.8 per 100,000 women and 7.2 to 15.4 per 100,000 men [3]. PBC is mainly diagnosed between ages 35-60 and predominantly seen in women compared to men with a sex ratio of 9:1 [4]. According to Ludwig's and Scheuer's classification, PBC is divided into four stages based on histology, with Stage 4(cirrhosis) representing a poorer prognosis [5].

The etiology of PBC is still elusive, although genetics and environmental factors could play a role. The clinical presentation of PBC can range from asymptomatic to liver cirrhosis. Other symptoms include pruritus, keratoconjunctivitis sicca, xerostomia, fatigue, xanthelasma, hyperpigmentation of the skin, jaundice. Some patients with PBC are also diagnosed with a concomitant autoimmune disease proposing the involvement of genetics and immune abnormalities [6]. Presently, there are only two FDA-approved medical treatments of PBC, which are ursodeoxycholic and obeticholic acid. In addition, there is a shortage of clinical studies evaluating the presentation of PBC in males with concomitant type 1 diabetes due to its low incidence compared to the preponderance of PBC in females. Therefore, we present a case of primary biliary cholangitis stage 2 in a young male with concomitant autoimmune disease (type 1 diabetes mellitus).

## Case presentation

A 40-year-old Hispanic male with a prior medical history of fatty liver disease, type 1 diabetes mellitus, and hypertension was referred to the clinic due to an elevated liver function test (LFT). The patient presented with complaints of fatigue, dry skin, and pruritus that started almost two years prior. He described worsening intensity of pruritus in warm environments. He stated he had tried numerous anti-itch ointments but only found little relief when using lotion with an active ingredient of methanol 0.15%. The patient disclosed an episode of bilateral lower extremity edema that occurred four months ago, which resolved without any medical intervention. He denied any history of dry eyes, jaundice, abdominal pain, and weight loss. The patient's medication included aspirin, novolog, lantus, vitamin D, and lisinopril. He had no prior surgeries. He denied any family history of chronic liver diseases and had no known allergies. He stated he drank alcohol occasionally. The patient denied tobacco or any illicit substance use.

On physical examination, his vitals were as follows: blood pressure was 129/75 mmHg, respiratory rate was at 18 breaths per minute, heart rate was 87 beats per minute, the temperature was 36.3°C (97.3°F), weight was 72.6 kg, height was 167.6 cm, and body mass index was 25.82 kg/m^2^. The patient was alert, oriented, and in no acute distress. The rest of the clinical examination revealed no skin hyperpigmentation, no jaundice. On palpation, no peripheral edema was noted, the abdomen was soft, non-tender, non-distended, and no palpable masses or hepatosplenomegaly was noted. His Hemoglobin A1c was 9.5%, and he stated that home glucose levels range between 40-178 mmol/L.

**Figure 1:**
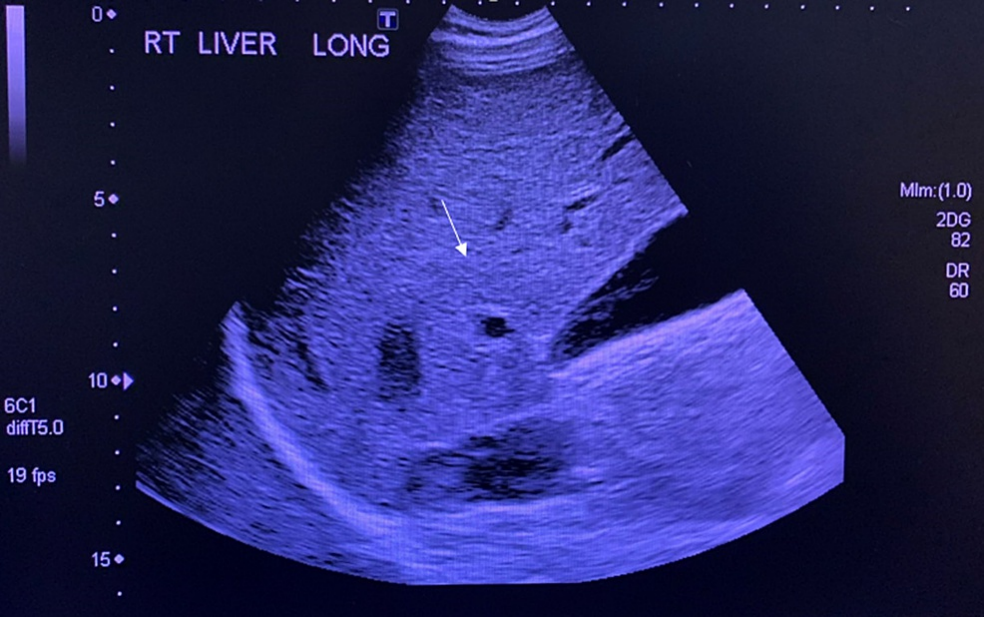
Ultrasound of the liver suggestive of fatty infiltration. Arrow pointing at a slight, diffusely increased echogenicity of a moderately enlarged liver which is a non-specific finding but can be seen in the setting of fatty infiltration.

**Figure 2:**
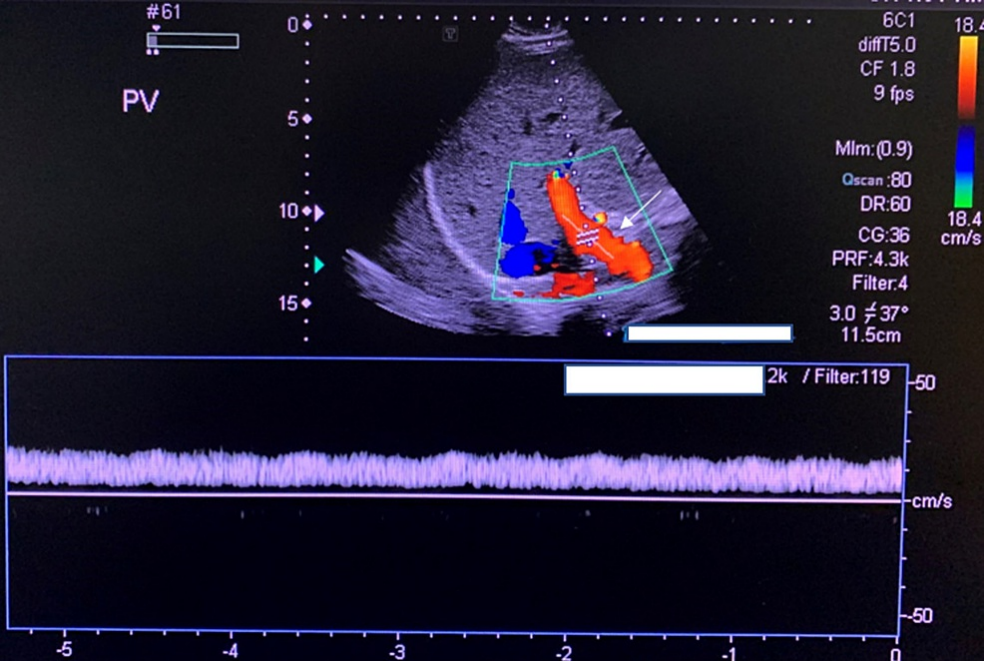
Ultrasound with doppler of the portal vein. The arrow points to the portal vein which demonstrated normal flow and direction of blood on Doppler.

**Figure 3:**
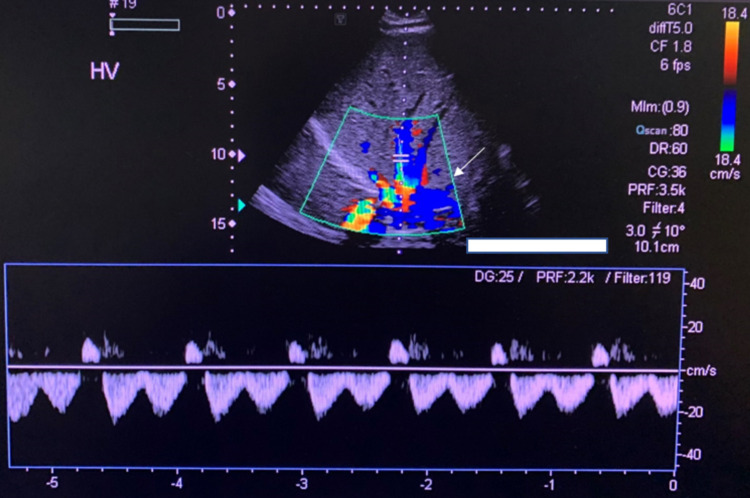
Ultrasound with Doppler of the hepatic vein. Arrow points to the hepatic vein which revealed a normal flow and direction of blood on doppler

**Figure 4:**
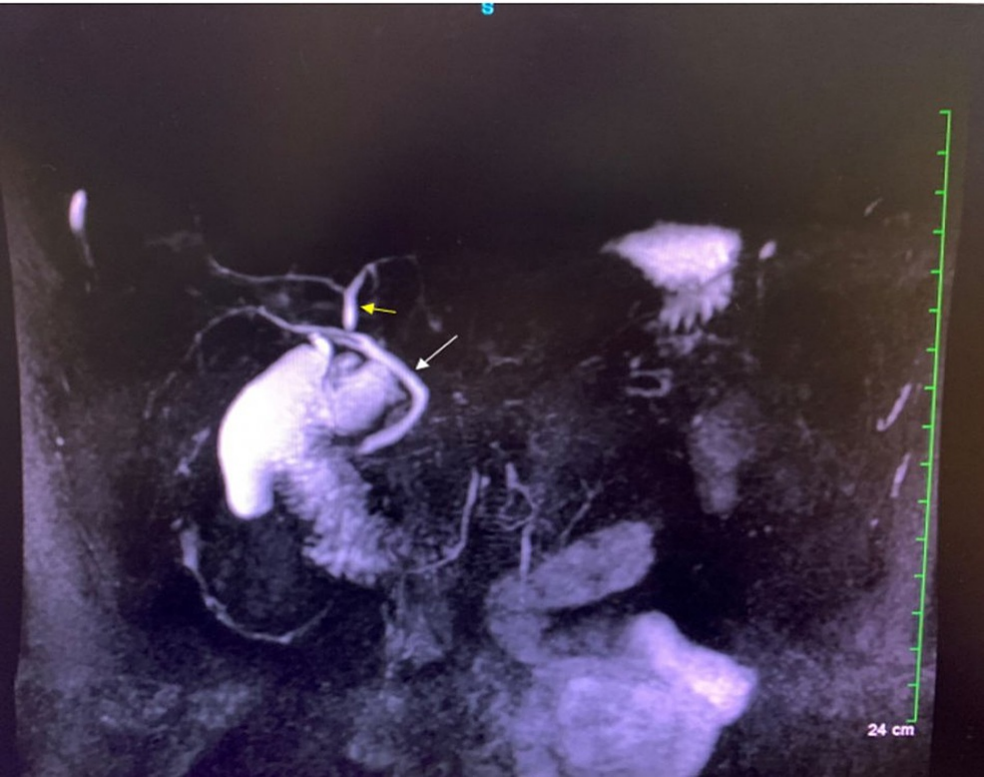
Magnetic resonance cholangiopancreatography revealing normal findings. There is no evidence of common biliary duct dilation (white arrow). Yellow arrow points at normal hepatic ducts. No stones are seen within the gallbladder and common bile duct.

The results of his LFT were as follows: alkaline phosphatase 1,366 IU/L, alanine transaminase 244 IU/L, aspartate transaminase 183 IU/L, gamma-glutamyltransferase 727 IU/L, total bilirubin 0.8mg/dL, direct bilirubin 0.25 mg/dL, and Albumin 3 g/dL. Ultrasound with Doppler performed revealed slight, diffusely increased echogenicity of a moderately enlarged liver consistent with fatty infiltration (Figure 1). There was no demonstrable focal intrahepatic defect, cholelithiasis, intrahepatic biliary dilation, or extrahepatic biliary dilation. In addition, there was a normal directional portal (Figure 2) and hepatic venous flow (Figure 3). A subsequent MRCP to rule out primary sclerosing cholangitis was unremarkable (Figure 4). Autoantibody testing showed a positive Antimitochondrial antibody level of 118.5 units and a negative Anti-smooth muscle antibody. He was prescribed Ursodiol 300mg three times a day with meals, cholestyramine 4 g twice a day for pruritus, and referred for liver biopsy for further evaluation and definitive diagnosis.

**Figure 5:**
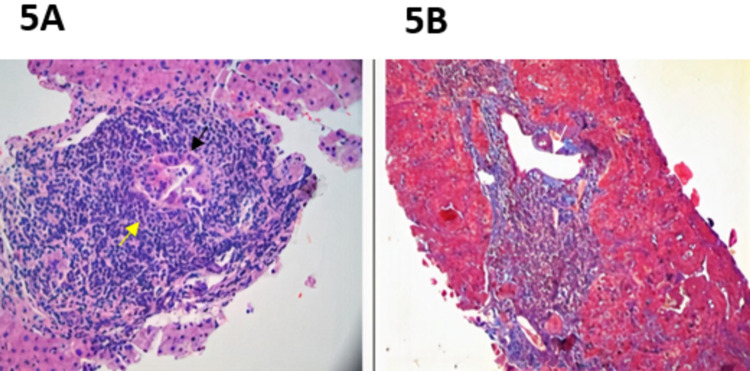
Liver biopsy consistent with primary biliary cholangitis. A: Biliary pattern injury with mild to moderate chronic lymphoplasmacytic portal inflammation (yellow arrow), bile duct injury, ductular reaction, and some bile duct loss (black arrow) consistent with primary biliary cholangitis. B: White arrow points to normal portal veins. No granuloma, steatosis, or cholestasis is present.

The liver biopsy had a microscopic description of an intact architecture; several portals were mild to moderately expanded with lymphocytes, plasma cells, and few eosinophils with interface activities and sparse lobular lymphocytic inflammation with focal hepatocyte dropout. In addition, focal bile duct injury, ductular reaction, and some bile duct loss were present (Figure 5). These findings are consistent with primary biliary cholangitis. The biopsy result was discussed at length with the patient. Online educational resources regarding PBC were provided to the patient. Also, the patient was educated on the importance of regular blood glucose monitoring and medication compliance.

At a follow-up visit, six weeks subsequent to the diagnosis of PBC, the patient stated that his pruritus was well controlled with cholestyramine. However, he reported side effects of decreased appetite and nausea with cholestyramine use. In addition, the patient noted no improvements in his symptoms of fatigue and dry skin. The physical examination was unremarkable, with no changes from prior examinations. A repeat liver function test was performed, and the results were as follows: Alkaline Phosphatase 1,275 IU/L, Alanine transaminase 222 IU/L, Aspartate transaminase 156 IU/L, Albumin 3.4 g/dL, and direct bilirubin 0.23 mg/dL. The patient was encouraged to continue his current medication and scheduled a six-week follow-up to monitor his disease progression and other symptoms. 

## Discussion

Primary biliary cholangitis is a female predominant autoimmune disorder characterized by chronic inflammation, granulomatous destruction, and fibrosis of the intrahepatic bile ducts. This autoimmune disorder is mainly mediated by T cells. The exact etiology and pathophysiology involving PBC in both males and females remain unclear. However, several factors such as genetics, sex hormones, micro-organisms, and the environment have proven to play a role in PBC [7]. Several types of research are done to elucidate the cause of the low occurrence of PBC in males compared to females. The difference in the immune system is one proposed theory. A study was done to compare the T lymphocytes and antigen-presenting cells (APC) between males and females; the study revealed that females tend to exhibit a higher increase in CD4 T cell count and increased immune response after immunization which can explain the higher incidence of autoimmune diseases seen in females. It also revealed that in healthy males, testosterone plays a huge role in reducing immunoglobulin production by peripheral blood cells [8]. Regardless of the proposed etiological differences seen between males and females, the characteristic antibody involved in PBC is called anti-mitochondrial antibody (AMA), which selectively targets the epithelium of the small intrahepatic bile ducts. AMA can be detected in 95 % of patients with PBC and is commonly detected via enzyme-linked immunosorbent assay (ELISA). As the name of the antibody implies, it targets mitochondrial enzyme complexes such as the E2 component of the pyruvate dehydrogenase complex (PDC-E2), E2 component of the branched chain 2-oxo-acid dehydrogenase complex (BCOADC-E2), and the central E2 component of 2-oxoglutarate dehydrogenase complex (OGDC-E2) [4]. AMA easily accesses these mitochondrial enzyme complexes due to the apoptosis of the epithelial cells of the bile duct. This apoptosis is due to the downregulation of bcl-2 (anti-apoptotic protein) [9]. A study conducted by Nalbandian and colleagues revealed that both men and women produced high levels of AMA with similar antigenic specificity and frequency of reactivity to the three main mitochondrial enzyme complexes [10]. Infections with bacteria and viruses is another proposed theory that explains the etiology of PBC in males. A study revealed that some bacterial and viral proteins had been noted to contain molecular mimicry peptides to PDC-E2 [11], with males exhibiting an increased inflammatory response to infectious organisms [8].

Risk factors such as smoking, alcohol, hair dye use, nail polish use, family history of PBC, recurrent UTI have been indicated to play a role in the onset of PBC. Several studies done to evaluate the difference in the risk factor of PBC between males and females have not demonstrated any risk factor predominant to males only [6]. However, occupational exposure, e.g., to coal and steel, has indicated an increase in PBC diagnosis amongst men, although this observation needs to be studied further [12].

Primary biliary cholangitis can exist with other autoimmune conditions which may arise from several systems such as endocrinology, pulmonary, rheumatology, and gastroenterology. Based on the co-existence of these disorders, it could be deduced that these conditions may share similar genetic predispositions. A study was done on 1,032 people to evaluate the presence of other comorbid autoimmune diseases in PBC. It was observed that other autoimmune conditions were present in 32% of cases compared to 13% noted in the control group. Raynaud's syndrome and Sjogren's syndrome were the most recurring among PBC patients [6]. However, in our case report, we presented a male with PBC and insulin-dependent diabetes mellitus (type 1). The co-existence of these two autoimmune conditions is very rare hence the presence of limited research on how type 1 diabetes mellitus (DM) plays a role in PBC; however, recent studies propose that their connection is due to a similar pathway involving protein compounds with hydrodynamic properties of human endogenous retroviruses. This same retrovirus is involved in the onset and progression of type 1 diabetes [13]. A study was done to reveal the influence of diabetes mellitus on the natural progression of PBC using non-invasive scores to predict fibrosis. The non-invasive scores were higher in PBC patients with DM with a fibrosis-4 score of 4.08 than a lower fibrosis-4 score of 3.21 in PBC patients without DM. In summary, patients with PBC and co-morbid diabetes mellitus developed cirrhosis at an increased rate (62.2%) versus 42% in PBC-only patients. Based on these statistics, it can be proposed that effective control of DM may slow down the progression of PBC to cirrhosis [14].

PBC can cause devitalizing symptoms such as fatigue, intermittent pruritus, jaundice, xanthelasma, xanthoma, keratoconjunctivitis sicca, xerosis, and eventually cirrhosis, liver failure, and death. In addition, patients with PBC may exhibit symptoms seen in other autoimmune conditions such as thyroid disorders or Sjogren's syndrome [6]. Most patients, however, are asymptomatic when diagnosed with PBC; these asymptomatic patients are usually referred to the hepatologist when liver enzyme abnormalities are noted on routine blood work.

**Figure 6:**
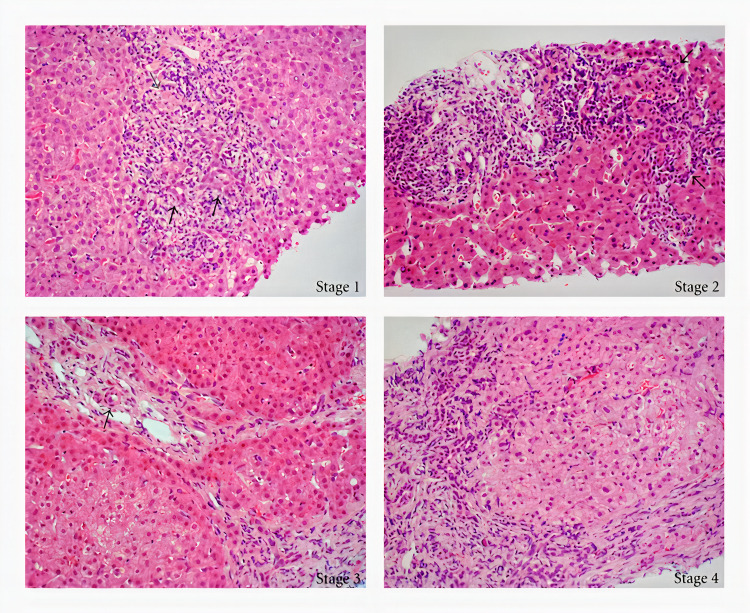
In stage 1; black arrows reveal duct-centered inflammation showing chronic nonsuppurative destructive cholangitis. The grey arrow reveals a tiny granuloma. The black arrows in stage 2 reveal portal enlargement with bile ductular reaction and inflammatory cell infiltration. Stage 3 reveals fibrous scarring bridging portal tracts with occasional foci of bile duct loss. The arrow indicates that no bile duct is identified around an artery. Stage 4 shows a cirrhotic transformation. Sex Differences Associated with Primary Biliary Cirrhosis - Scientific Figure on ResearchGate. Available from: https://www.researchgate.net/figure/Histological-staging-in-a-representative-case-of-a-patient-with-primary-biliary-Stage-1_fig2_225302082 [accessed 20 Jun, 2021]

Diagnosing PBC begins initially with history and physical followed by focused laboratory tests including aspartate aminotransferase (AST), alanine aminotransferase (ALT), Total bilirubin, alkaline phosphatase (ALP), gamma-glutamyl transferase (GGT), which will help confirm the liver origin of elevated serum ALP. The level of bilirubin indicates a valid predictor of prognosis in PBC. Hyperbilirubinemia, especially when it co-exists with low albumin, decreased platelets, and increased international normalized ratio (INR), suggests the development of advanced liver disease. An important serologic marker that helps to support the diagnosis of PBC is an elevated anti-mitochondrial antibody (AMA) level which is usually determined by indirect immunofluorescence (IIF) or enzyme-linked immunosorbent assay (ELISA). A positive result occurs when AMA is greater than or equal to 1:40 with ELISA or greater than or equal to 25 with IIF. Despite AMA levels being a hallmark for diagnosing PBC, its presence is not enough to diagnose PBC; hence further evaluation is needed. Liver imaging in the form of ultrasound, computerized tomography (CT) scan, Magnetic resonance cholangiopancreatography (MRCP) are additional diagnostic modalities that can help in further diagnosis of PBC and rule out differential diagnosis such as choledocholithiasis, malignant biliary obstruction, primary sclerosing cholangitis, infiltrative liver diseases. In situations where all above mentioned diagnostic modalities are inconclusive, but there are high clinical suspicions, liver biopsy is usually the last resort to help confirm PBC and diagnose co-morbid liver disease. A liver biopsy on histology reveals inflammation of the bile ducts, characterized by destruction, proliferation, and fibrosis of bile ductules, intraepithelial lymphocytes, and periductal granulomas [5,7]. In summary, the diagnosis of PBC is made when two of the following three criteria are met: an increase in alkaline phosphatase (ALP), a Positive serology test including AMA, sp100, or gp210 if AMA is negative and histologic evidence of PBC [15]. Liver biopsy was historically used to stage PBC into four, using the Ludwig and Scheuer scoring systems [5]. Stage 1 reveals portal inflammation that is confined to the portal areas and normal-sized portal triad. Stage 2 reveals an enlarged triad with a portal and periportal inflammation. Stage 3 reveals bridging fibrosis of the liver septa. Finally, stage 4, which indicates the poorest prognosis, reveals cirrhosis (Figure 6).

The treatment of PBC mainly encompasses medications that are anti-cholestatic. Ursodeoxycholic acid (UDCA ) dosed at 13-15 mg/kg/day was the first drug to be approved for PBC treatment in 2004. UDCA is a natural, non-toxic bile acid that increases the flow of bile from the hepatocyte, prevents further injury to the hepatocytes by inhibiting apoptosis of bile ducts and the production of toxic bile acids [16]. UDCA has been proven in several studies to improve the liver function tests (LFT) in patients with PBC. A study was done on 15 patients diagnosed with PBC to evaluate the reduction in LFT. In the two years following up, the levels of alkaline phosphatases, alanine aminotransferase, gamma-glutamyl transpeptidase, and bilirubin were reduced [16]. Recent studies have also proved the use of long-term UDCA therapy to reduce liver transplants as it helps slow down the progression of PBC to cirrhosis [17]. Obeticholic acid is another FDA-approved medication for PBC. It is an anti-cholestatic agent usually used as a single therapy in PBC in patients who have developed an intolerance to UDCA. Obeticholic acid can also be used in combination with UDCA to treat PBC refractory to UDCA [18].

Symptomatic treatment is also vital in the management of PBC. The first and second-line agents used to treat pruritus in PBC are cholestyramine (4-12 g/d) and rifampin, respectively. Medications that block histamine pathways do not resolve the itch in PBC. Due to the alteration of bile flow in PBC, there is malabsorption of fat and fat-soluble vitamins, which can present with symptoms of diarrhea, so supplementation of vitamins K, A, D, and calcium should be considered. There are other medications that have been proven to be beneficial in some ways in PBC management. Medications like budesonide, immunosuppressants such as azathioprine, cyclosporine, methotrexate etc. although further studies are needed to establish the role of these medications in the management of PBC [19].

The final resort in the management of PBC is liver transplantation. This procedure is carried out in patients exhibiting signs of end-stage liver failure such as ascites, jaundice, refractory encephalopathy, recurrent spontaneous bacterial peritonitis etc. [19]. The model for end-stage liver disease (MELD) score, which ranges from 6 to 40, evaluates creatinine, International normalized ratio (INR), and bilirubin to determine the allocation of a liver to patients on the transplant list. The Pittsburgh Series, based on the mayos model, proposes that survival in PBC patients is improved after transplantation [20].

Based on the pathogenesis and pathophysiology of PBC, patients are at risk of complications such as osteoporosis, portal hypertension, esophageal varices, and hepatocellular carcinoma. Therefore prophylactic measures such as Dual-energy X-ray absorptiometry (DEXA) scan, Esophagogastroduodenoscopy (EGD), and liver ultrasound should be performed routinely [19].

## Conclusions

Due to the rarity of primary biliary cholangitis with concomitant type 1 diabetes in men, the exact etiology and pathophysiology of the diagnosis are not familiar. Even though very little is known about primary biliary cholangitis in men, current research suggests no significant difference can be noted in women diagnosed with PBC. The biochemical, immunological, and histological characteristics of PBC between males and females show several similarities. However, further research is needed to understand clinical courses, disease associations, and long-term prognosis in males with primary biliary cholangitis, especially with concomitant type 1 diabetes.
